# Relationship between child survival and malaria transmission: an analysis of the malaria transmission intensity and mortality burden across Africa (MTIMBA) project data in Rufiji demographic surveillance system, Tanzania

**DOI:** 10.1186/1475-2875-13-124

**Published:** 2014-03-28

**Authors:** Susan F Rumisha, Thomas A Smith, Honorati Masanja, Salim Abdulla, Penelope Vounatsou

**Affiliations:** 1 Swiss Tropical and Public Health Institute, Socinstrasse 57, 4051 Basel, Switzerland; 2 University of Basel, Petersplatz 1, 4051 Basel, Switzerland; 3 Ifakara Health Institute, PO Box 78373, Dar es Salaam, Tanzania; 4 National Institute for Medical Research, PO Box 9653, Dar es Salaam, Tanzania

**Keywords:** Child mortality, EIR, Time varying covariates, Mortality-transmission relation, DSS, Malaria, MTIMBA

## Abstract

**Background:**

The precise nature of the relationship between malaria mortality and levels of transmission is unclear. Due to methodological limitations, earlier efforts to assess the linkage have lead to inconclusive results. The malaria transmission intensity and mortality burden across Africa (MTIMBA) project initiated by the INDEPTH Network collected longitudinally entomological data within a number of sites in sub-Saharan Africa to study this relationship. This work linked the MTIMBA entomology database with the routinely collected vital events within the Rufiji Demographic Surveillance System to analyse the transmission-mortality relation in the region.

**Methods:**

Bayesian Bernoulli spatio-temporal Cox proportional hazards models with village clustering, adjusted for age and insecticide-treated nets (ITNs), were fitted to assess the relation between mortality and malaria transmission measured by entomology inoculation rate (EIR). EIR was predicted at household locations using transmission models and it was incorporated in the model as a covariate with measure of uncertainty. Effects of covariates estimated by the model are reported as hazard ratios (HR) with 95% Bayesian confidence interval (BCI) and spatial and temporal parameters are presented.

**Results:**

Separate analysis was carried out for neonates, infants and children 1–4 years of age. No significant relation between all-cause mortality and intensity of malaria transmission was indicated at any age in childhood. However, a strong age effect was shown. Comparing effects of ITN and EIR on mortality at different age categories, a decrease in protective efficacy of ITN was observed (i.e. neonates: HR = 0.65; 95% BCI:0.39-1.05; infants: HR = 0.72; 95% BCI:0.48-1.07; children 1–4 years: HR = 0.88; 95% BCI:0.62-1.23) and reduction on the effect of malaria transmission exposure was detected (i.e. neonates: HR = 1.15; 95% BCI:0.95-1.36; infants: HR = 1.13; 95% BCI:0.98-1.25; children 1–4 years: HR = 1.04; 95% BCI:0.89-1.18). A very strong spatial correlation was also observed.

**Conclusion:**

These results imply that assessing the malaria transmission-mortality relation involves more than the knowledge on the performance of interventions and control measures. This relation depends on the levels of malaria endemicity and transmission intensity, which varies significantly between different settings. Thus, sub-regions analyses are necessary to validate and assess reproducibility of findings.

## Background

In sub-Saharan African countries about 20% of all deaths occurring in under-fives are attributed to malaria [1-4]. The disease contributes significantly in morbidity and mortality burden at all ages and is a main confounder of other conditions and causes of deaths in children such as low birth weight, malnutrition and anaemia [5,6]. Recently, a decline in child mortality has been observed in most developing countries [4,7,8]. The drop in mortality is partly associated with success in interventions and control strategies targeting malaria transmission, such as insecticide-treated nets (ITNs) and efficacious anti-malarial drugs [9-12]. For instance in the Rufiji demographic surveillance system (DSS), changing of the first-line drug for malaria treatment from chloroquine to sulphadoxine-pyrimethamine and increasing coverage of ITNs were followed by a sharp decline in mortality and malaria transmission [13,14]. The traces on the possible relationship between mortality and fluctuation in malaria transmission exists, however, lack of vital registration in developing countries, unreliable information on specific causes of deaths, problems related to disease diagnosis e.g. malaria, lack of appropriate data to track changes in malaria transmission complicate efforts to understand the transmission-mortality relationship [15-18]. Malaria control programmes have usually used all-cause mortality as an essential indicator in under-fives [15,19,20] yet data on transmission remains a concern [21]. Progress towards malaria eradication as the long-term vision of Roll Back Malaria (RBM) partnership, requires accurate knowledge of the transmission-mortality relation [15,21-23].

Discrepant results that might be related to higher levels of indirect mortality attributable to malaria have been observed in various attempts to relate transmission and mortality. In a review article, Smith *et al.*[20] found an increase in infant mortality rate with increase in entomological inoculation rate (EIR) in Africa. However, Gemperli [22] linked the demographic and health surveys (DHS) and mapping malaria risk in Africa (MARA) databases to assess the effect of malaria transmission on mortality, and found no clear relationship. In a study conducted in western Kenya, no difference in mortality rates could be observed between villages with and without ITN intervention [24]. These conclusions are based on reviews and/or analyses of aggregated data from studies conducted at different times (periods), regions and designs, which might not be directly comparable. In addition, many of these studies were not specifically designed to assess the mortality attributed to malaria [25]. In malaria-endemic areas, mortality is influenced not only by disease (malaria) transmission but also by factors related to poverty, cultural aspects, literacy, control interventions, and health systems performance [26-30] and it is usually challenging to take these factors into account. High prevalence of HIV in some regions adds a substantial challenge [31,32].

The malaria transmission intensity and mortality burden across Africa (MTIMBA) project initiated by the INDEPTH Network [31-33] was designed specifically to assess the malaria transmission-mortality relationship. Integrated within the DSS which routinely monitors mortality, causes of death and other demographic parameters [34-36], the MTIMBA project collected biweekly entomological data at a large number of georeferenced household locations, using standardized methodology for a period of three years [33,37]. The MTIMBA database has the epidemiological information and statistical strength required to study the above relationship. However, data characteristics such as spatiotemporal correlations over large number of locations and lack of appropriate statistical methodologies delayed the data analysis to date. Recent approaches in modelling large geostatistical data [38] approximate the spatial process from a subset of locations. These methods have been applied to model the MTIMBA entomological data in Rufiji DSS in Tanzania [39] and estimate monthly surfaces of the EIR malaria transmission measure during the three years of the project.

**Figure 1:**
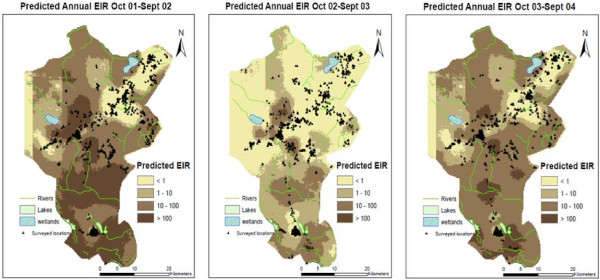
Villages of the Rufiji Demographic Surveillance System, Tanzania (Unpublished data: John S. Noronha, Ifakara Health Institute, Tanzania.

**Figure 2:**
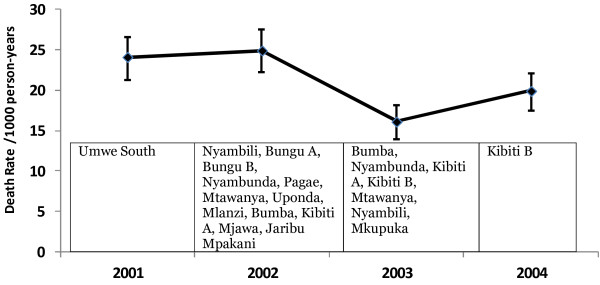
**Temporal patterns (annual) variation of Child Mortality in Rufiji DSS with villages identified as possible Spatial mortality clusters**[13]**.**

The spatial-temporal patterns of transmission intensity [39] may introduce space and time variations in mortality rate. Shabani *et al.*[13] provided evidence of space and time correlation in mortality within the Rufiji DSS area by identifying spatial–temporal mortality clusters in the DSS (Figure 1; Figure 2) using the spatial scan statistics [40]. In this study, the Rufiji DSS-mortality databases are linked to EIR estimates to assess the relationship between malaria transmission and all-cause mortality in children less than five years old. The analysis is conducted using Bayesian geostatistical and temporal regression models applied on the mortality outcome, considering EIR as predictor and adjusting for malaria control interventions. The EIR is predicted from a spatiotemporal transmission model and the prediction uncertainty is incorporated during estimation of mortality risk [39].

## Methods

### Study area

Rufiji District is one of the six districts of the Coastal Region in the southeast part of Tanzania, with a population size of about 182,000 inhabitants. The Rufiji DSS is located in the Rufiji District (7.47°-8.03° south latitude and 38.62°-39.17° east longitude). The Rufiji DSS covers an area of 1,813 sq km with 85,000 inhabitants under surveillance [33]. The population density is 46 people per sq km and the average household size is five people [41]. The major causes of mortality in the Rufiji DSS include acute respiratory infections, tuberculosis, acquired immunodeficiency syndrome (AIDS), perinatal causes, and malaria [13]. Malaria is endemic and seasonal throughout the region. Higher transmission occurs during and shortly after the rains (March to June). Prevalence of malaria was 28% in 2002 and 20% in 2004, which is an approximately 28% reduction in a period of two years. Considering the higher amount of rain in the year 2004 as compared to 2002 which was relative a dry year, more malaria would have expected at later. The reduction indifferently suggests presence of underlying factors related to changes in malaria prevalence [33].

### Mortality, demographic and malaria intervention data

Child all-cause mortality data were obtained from the Rufiji DSS database for the period of the MTIMBA project (i.e., October 2001-September 2004). Individual-specific information extracted from the DSS database includes date of birth, start and exit from the study, age, sex, and vital status (1 if death occurred during the study period and within the DSS and 0 otherwise). Other information such as ITN possession, socio-economic status [33], travel time to health facilities, and altitude were taken from other sources, such as district health plans, reports and linked to the mortality database (Table 1). Time at risk (person-days) contributed by each child was calculated until exit. Exit from the study was due to migration (outside the DSS area), death or end of the study. In a case where a child migrated to a different household location within the study area, time at risk was computed separately for new location and added to the total time at risk. The outcome of interest is the death status of an individual or total death for specific age groups. The mortality rates were expressed per 1,000 person-years (py).

**Table 1:** Number of individuals, deaths and locations after merging mortality database with entomological, socio-economic and malaria interventions databases

**Merged database**	**Unique locations**	**Individuals**	**Deaths counts (%)**
Mortality	14,847	27,049	831 (3.07%)
Entomological	11,631	23,905	768 (3.21%)
Socio-economic	9,574	20,341	651 (3.20%)
Malaria interventions	8,144	17,717	567 (3.20%)

### Malaria transmission data

**Figure 3:**
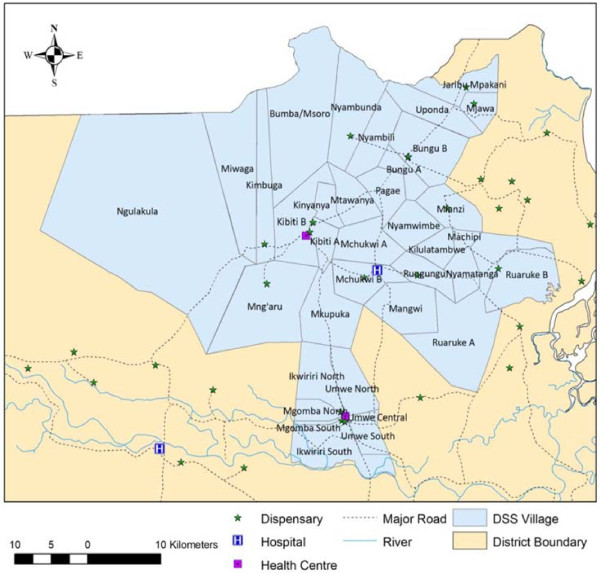
**Spatial temporal distribution of annual EIR in the Rufiji DSS**[39]**.**

The entomological data from the MTIMBA project were analysed using Bayesian geostatistical models to obtain EIR estimates at locations (households) and months where mortality data were available [40]. In particular, separate geostatistical and temporal logistic regression and negative binomial models were fitted to sporozoite rate and mosquito density data, respectively. Using Bayesian prediction (kriging) and environmental factors as predictors, EIR was subsequently estimated by the product of the sporozoite rate and the man biting rate (MBR) predicted from the above models at the household locations. MBR was calculated from the mosquito density estimates [42]. Details of this work are available in Rumisha [39]. Model parameter estimates and maps (Figure 3) indicate strong spatio-temporal patterns of EIR.

### Linking mortality with other databases

The mortality database included information on 27,049 children residing from 14,847 household locations. The entomological, socio-economic, malaria interventions and DSS vital statistics databases were linked using household geographical locations (latitude and longitude). The final dataset included 17,717 children from 8,144 households. About 45% of the household locations with mortality data were dropped during the merging process, however, the proportion of deaths remained similar (Table 1).

### Statistical analysis

Survival analysis using non-spatial Cox Proportional Hazards models was performed for different groups of child age (neonatals (0–28 days); post-neonatal (29 days-11 months); infants (0 days-11 months); children^1^ (0 days-59 months); children^2^ (29 days-59 months); and, children^3^ (12 months-59 months) to assess differences in mortality risks between the groups and decide whether separate analyses are required for each age subgroup. Mortality rates were calculated and compared between subgroups (Table 2). For the selected groups, bivariate models were fitted to assess potential non-linearity in the relation between EIR and mortality by considering the following transformations of EIR: i) categorical; ii) logarithmic (natural); and, iii) fractional polynomials of different orders. For the logarithm transformation, a Taylor series approximation for the exponential function was used to obtain mortality rates at zero level of transmission [43]. The Akaike Information Criterion (AIC) was used to assess the model performance and select the best model including the one assuming linearity [44]. The best model was the one with the smallest AIC value. These analyses were carried out in STATA v10 [45].

**Table 2:** Descriptive statistics on mortality at different age groups of child health, October 2001 to September 2004, Rufiji DSS

**Group**	**Age range**	n*	**Death counts**	**Person years**	**Mortality rate (95% CI) (per 1,000 py)**
1	Neonatal (0–28 days)	9,758	213	712	299.2 (265.71, 334.27)
2	Infants (0 days-11 months)	13,228	517	9,239	56.0 (51.35, 60.84)
3	Post-neonatal (29 days-11 months)	13,015	304	9,236	32.9 (29.36, 36.75)
4	Children^1^ (0 days-59 months)	27,049	831	44,286	18.8 (17.52, 20.07)
5	Children^2^ (29 days-59 months)	26,836	618	44,283	14.0 (12.88, 15.09)
6	Children^3^ (12 months-59 months)	26,539	314	44,150	7.1 (6.35, 7.94)

*including migration within the DSS area.

In addition, bivariate and multivariate time-dependent Proportional Hazards survival models with spatial and independent, village-level, random effects were fitted for selected age groups. These models were approximated by a pooled logistic regression [46,47] and included monthly temporal random effects. The spatial random effects were considered to derive from a zero-mean multivariate normal distribution [48] with covariance matrix assuming that spatial correlation decays exponentially with distance between villages. The temporal random effects were modelled by a first order autoregressive process. Following a Bayesian formulation, appropriate prior distributions for the parameters were adopted.

The time at risk were disaggregated and split by months to incorporate a time varying trend of EIR. Values of EIR (with natural logarithmic transformed) were then assumed to arise from a normal distribution with a mean and variance defined by median and standard deviation of the posterior predictive distribution, respectively. This allowed taking into account the measurement errors of predicted EIR when estimating the regression parameters. The mortality events were related with a one-month lag EIR. Other predictors considered included age, sex, socio-economic status, ITN ownership, first-line malarial drug, travel time to the health facility, and altitude. All geostatistical models were fitted in OpenBUGS version 3.0.3 (Imperial College and Medical Research Council; London, UK) [48,49]. Formulation and specification of the geostatistical model are given in Additional file 1.

### Ethical consideration

The study received ethical approval from the institutional review boards of the Ifakara Health Research and Development Centre and the Medical Research Coordination Committee of the National Medical Research Coordination Committee of National Institute for Medical Research, Tanzania (Reference number NIMR/HQ/R.8a/VOL.VIII, April 2000).

### Consent

Written informed consent was obtained from each study participants in the Rufiji DSS. Local leaders at village levels and staff of the Council Health Management Team were also informed at about the survey and how the data will be used [50].

## Results

### Mortality data and selection of age category for analysis

The complete mortality database included 27,049 children from 32 villages, which were followed up during the project period. The mean follow-up time was 1.6 years with a total time at risk of 44,286 py. Total individuals at the commencing of the study period were 15,377. Among these 2% (n = 315) were neonatal, 21% (3,207) post-neonatal, and 77.1% (11,855) were one to four years (59 months). A total of 8,528 children entered in the course of the study (via birth or in-migration) and 831 deaths were registered for the entire study period. The overall under-five mortality rate for the three-year period was 18.7 per 1,000 py. For year 1 (October 2001-September 2002), year 2 (October 2002- September 2003) and year 3 (October 2003-September 2004) of the study, the death counts (mortality rates per 1,000 py) were 321 (261.5), 237 (68.9) and 273 (7.1), respectively (bivariate analysis: p-value <0.001). The mean age at death was 2.36 years (±1.44). Almost a quarter (26%, n = 213) of all deaths occurred in children aged less than one month. The proportions of death in the post-neonatal age group (n = 304, 37%) and in children aged 12–59 months (n = 314, 37%) were very similar. Table 2 shows the number of children, numbers of deaths and mortality rates in different age groups.

The mortality rates declined with age. The highest rate was observed in neonates. There was a significant difference in mortality in the age categories were neonates were included or omitted in the calculations. Observe mortality presented by groups 2 *vs* 3 and 4 *vs* 5 in Table 2. These observations suggest separate analysis is needed such groups. In addition, mortality rates in children with and without post-neonatal (groups 5 and 6) differ significantly. Taking all these descriptions into consideration, three separate analyses were conducted for: i) neonates (0–28 days); ii) post-neonatal (29 days-11 months); and, iii) children^3^ (12 months-59 months).

### Exploratory analysis

The results of the exploratory analysis carried out on the final datasets including all covariates (i.e., 17,717 children and 567 deaths) are given in Table 3 and Table 4. EIR was categorized into five groups and mortality rates were estimated for each group. Bivariate analysis did not indicate strong associations of EIR with mortality, however, the mortality rates considerably decreased across age categories. Low mortality is observed in individuals with ITNs compared to those without (Table 3 and Table 4). The overall levels of ITN ownership across socio-economic status quintiles (first to fifth), were 0.0, 3.3, 23.8, 28.9 and 44.0%, respectively. These proportions indicate a significant relationship between ITN possession and levels of income.

**Table 3:** Mortality rate according to insecticide-treated nets possession, socio-economic status and EIR levels in the Rufiji DSS: neonates and infants

	**Neonates**			**Infants**		
**Variable**	**n**	**Deaths**	**MR (95% CI)**	**n**	**Deaths**	**MR (95% CI)**
EIR						
0	4,946	101	291.9 (244.5, 342.9)	7,852	132	27.1 (22.7, 32)
>0.0-1	809	18	367.3 (234.2, 517.1)	2,897	17	11.6 (6.8, 18.5)*
>1-10	1,323	20	238.1 (151.9, 343.5)	3,842	44	22.0 (16, 29.4)
>10-100	442	8	285.7 (132.2, 486.7)	1,280	13	20.2 (10.8, 34.3)
>100	34	2	1,000 (158.1, 1,032.4)	118	2	35.7 (4.4, 123.1)
ITN possession						
No	5,145	128	344.1 (295.9, 394.8)	6,801	177	40.1 (34.5, 46.3)
Yes	1,279	21	225.8 (145.5, 324.2)	1,731	31	27.9 (19.1, 39.4)
Socio-economic status						
Poorest	1,156	21	250.0 (161.9, 356.4)	1,560	44	44.0 (32.2, 58.7)
Very poor	1,351	37	377.6 (281.6, 481.2)	1,783	47	39.9 (29.5, 52.7)
Poor	1,514	42	381.8 (290.8, 479.3)	1,963	47	36.3 (26.8, 48)
Less poor	1,376	23	230.0 (151.7, 324.9)	1,841	41	34.9 (25.2, 47)
Least poor	1,027	26	351.4 (243.9, 471.1)	1,385	29	33.0 (22. 2,47)

*Significantly associated with mortality (bivariate analysis); MR = Mortality rate; n = person years.

**Table 4:** Mortality rate according to -treated nets possession, socio-economic status and EIR levels in the Rufiji DSS: children 1–4 years and total population

	**Children 1-4**			**All**		
**Variable**	**n**	**Deaths**	**MR (95% CI)**	**n**	**Deaths**	**MR (95% CI)**
EIR (infectious bites/person/year)						
0	14,486	159	7.8 (6.6, 9.1)	17,455	392	15.1 (13.6, 16.6)*
>0.0-1	7,525	26	2.6 (1.7, 3.8)	9,225	61	4.6 (3.5, 5.9)
>1-10	8,883	27	2.4 (1.6, 3.5)	10,768	91	6.2 (5, 7.6)
>10-100	3,243	14	4.1 (2.3, 6.9)	4,024	35	8.0 (5.6, 11.1)
>100	327	0	0.0 (0, 13.8)	417	4	11.5 (3.1, 29.3)
ITN possession						
No	12,195	183	10.6 (9.1, 12.2)	14,704	488	22.1 (20.2, 24.1)
Yes	3,169	43	9.4 (6.8, 12.7)	3,775	95	16.5 (13.3, 20.1)*
Socio-economic status						
Poorest	2,783	49	12.5 (9.3, 16.5)	3,345	114	22.8 (18.8, 27.3)
Very poor	3,237	57	12.2 (9.3, 15.8)	3,875	141	23.8 (20.1, 28)
Poor	3,496	45	9.0 (6.5, 12.0)	4,207	134	20.9 (17.5, 24.7)
Less poor	3,299	48	10.3 (7.6, 13.6)	3,971	112	18.9 (15.6, 22.7)
Least poor	2,549	27	7.5 (4.9, 10.8)*	3,081	82	17.9 (14.3, 22.2)

*Significantly associated with mortality (bivariate analysis); MR = Mortality rate; n = person years.

The overall poorest/least-poor mortality ratio was 1.49 and the ratios were 0.71, 1.33, 1.66 for neonate, infants and older children, respectively. This suggests that (except for the neonates) within this region, children living in poorest families have on average 50% higher risk of dying than those living in better-off families. Higher mortality rate was marked in households with many members (>ten) than those with fewer individuals (≤five) (bivariate analysis: p-value <0.001). Differences in the mortality by family size could be highly influenced by the variation in the socio-economic status of the families, which differentiate care seeking patterns, alter ITN possession and utilization. Considering the bi-directional and structural link between these factors only ITN possession was included in the models. Travel time to health facilities and altitude were not significant in bivariate analysis, hence not included in final models.

### Model-based results

The natural logarithmic transformation of EIR provided the lowest AIC value and was used for analysis. Results of parameters estimated from multivariate spatial-temporal models for all groups, i.e., neonates, infants and children are described in Table 5. These include hazard ratio (HR) for predictors, spatial and temporal parameters.

**Table 5:** Parameter estimates obtained from Bayesian spatial-temporal models on neonates, infants and older children survival in the Rufiji DSS

	**Neonates (0–28 days)**	**Infants (1–11 months)**	**Children (12–59 months)**
**Variable**	**HR (95% BCI)**	**HR (95% BCI)**	**HR (95% BCI)**
**Covariates**			
Age	**0.79 (0.77, 0.82)**^**†**^	**0.92 (0.88, 0.96)**^**‡**^	**0.97 (0.96, 0.98)**^**‡**^
ITN use	0.65 (0.39, 1.05)	0.72 (0.48, 1.07)	0.88 (0.62, 1.23)
EIR (natural log)	1.15 (0.95, 1.36)	1.13 (0.98, 1.25)	1.04 (0.89, 1.18)
**Other parameters**			
Spatial range (in km)	56.32 (16.12, 82.15)	55.81 (17.2, 82.08)	54.62 (15.68, 82.06)
Spatial variance	0.28 (0.13, 0.74)	0.29 (0.13, 0.80)	0.30 (0.13, 0.83)
Temporal variance	0.22 (0.11, 0.57)	0.23 (0.11, 0.52)	0.26 (0.12, 0.70)
Non-spatial variance	0.22 (0.11, 0.46)	0.21 (0.11, 0.46)	0.20 (0.10, 0.44)
Autocorrelation	0.32 (−0.67, 0.98)	0.45 (−0.51, 0.94)	0.99 (−0.09, 1.00)

^†^Age in days; ^‡^Age in months.

In all categories, age is negatively related with the odds of dying and this is more prominent for the neonates (HR = 0.79, 95% CI: 0.77, 0.82). No significant association was obtained between mortality and ITN possession or malaria transmission intensity. Nevertheless, comparing very young children and older ones, a decrease in the protective effect of ITN (i.e., 35, 28 and 12% for neonates, infants and older children, respectively) was observed. Reduction in the odds of mortality with levels of malaria transmission intensity was indicated (i.e., 15, 13 and 4% for neonates, infants and older children, respectively) (Table 5). Similarly, pooled analysis (combining all age categories) did not indicate significant effect of neither EIR nor ITNs on mortality. The spatial range was similar in all groups and showed a strong correlation with a wide interval (covering almost the entire DSS area).

## Discussion

Recent health statistics report substantial reductions in mortality rates in some areas in the sub-Saharan Africa (SSA) region [12,51], and a drop in malaria infection is among the main factors that have been linked with this decline in the mortality. However, the relationship between child mortality and malaria transmission is not well understood [10,15,22]. This study assessed the relationship between malaria transmission and all-cause child mortality by linking the Rufiji DSS-mortality events with the malaria transmission database from the INDEPTH-MTIMBA project. This project is among the few initiatives aiming to understand the longitudinal effect of intervening malaria transmission on mortality in children and adults in different, malaria-endemic areas in SSA [20,31]. The intensity of malaria is measured by the EIR predicted at households that are routinely monitored for vital events, which created the opportunity for precise estimation of the exposure and quantification of the relationship. Separate analyses were conducted for neonatal, infants and older children. The uncertainty of the EIR estimates is incorporated by including the measurement error of the predicted EIR. The models were adjusted for age, the effect of malaria-related control strategies and took into account the spatial-temporal correlations.

This study found rather small relationships between estimated malaria transmission and child mortality, that were not statistically significant, although ITNs appeared to have a large impact, suggesting that malaria was a major cause of death in this population. A number of other malaria and interventions were routinely and effectively implemented. So the lack of an impact of malaria exposure could be a consequence of prompt and effective treatment averting many of the deaths. This could make it difficult to capture the actual transmission-mortality association within these settings [18,52]. It was clear that there was a clear downward trend of the effect of transmission with age which may be an effect of the cumulative malaria exposure [53]. High cumulative exposure reduces the risk of infection especially in older children [54,55].

A reduction on the protective efficacy of ITNs with age was indicated although the levels of ITN possession (and probably utilization) were very similar for the three subgroups. This observation might be associated with the acquisition of malaria immunity (which increases with age), or with behavioural change in older children [56]. It is expected that ITNs reduce the exposure of an individual to mosquitoes and hence reduce the chance of malaria transmission. Therefore, at early stages of life ITNs are beneficial as they lead to less maternal malaria and protect children with low (or no) immunity. With time children build up immunity and given that malaria prevalence is low, the effect of ITNs on their death risks becomes minimal, and factors other than malaria drive mortality in these children [57].

The low excess risks associated with malaria exposure could also be a consequence of transient effects due to changes in malaria transmission during the study period. A significant drop in mortality was observed in the second and third year of the study period. Factors related to improvement in the health services, access to care and food security could explain some of this the decline [51,58]. However, a two-thirds reduction of households with high malaria transmission occurred over the same period [39]. This could well have resulted in a shift in the maximum incidence of malaria mortality to older children, as mortality patterns change from those of a high transmission area to those expected in areas with intermediate transmission [59-61]. Monitoring of malaria mortality in different age groups over a longer period of time is required to evaluate the time courses of such reductions.

In providing estimates of how all-cause mortality varies with EIR, the models do not distinguish between direct and indirect malaria -related deaths. The other major causes of death in Rufiji include pneumonia, diarrheal disease, malnutrition or HIV/AIDS [31], all of which interact biologically with malaria, and so the underlying cause of many of the malaria-related deaths could be one of these conditions. Correlations between malaria exposure and mortality unrelated to malaria are captured by the effects of socio-economic status, which is known to be inversely related to malaria infection in Rufiji [62]. Poverty leads to poor access to care and more exposure to diseases, resulting in higher risk of death. The higher mortality risk observed for households with more members could also be related to the association between family size and income [63,64].

The spatial correlation was estimated at village level and the spatial range of more than 50 km was obtained. Among factors related to spatial differences in child mortality are bio-demographic factors, such as socio-economic status, maternal characteristics, place of birth and birth order [65,66]. Recent studies conducted in Western Africa [67] and in the same DSS area [32] reported a significant effect of socio-economic inequality and mothers education in child mortality. Both of these factors affect household and individual care seeking behaviours which are at the base of the cause-of-death in children. Limited access to some of these data at the needed spatial resolution at the time of analysis hinders incorporation into the analysis. On the other hand, some risk factors are not expected to differ much within the DSS area, which results to demonstration of wide dependency on occurrence of vital events. The missing data that could not be used for analysis due to lack of household-specific geographical coordinates might cost interpretation on the linkage between malaria transmission and child mortality, however, results from bivariate models which were fitted at different stages of data merging process showed similar pattern of the transmission-mortality relationship.

This study relates vital events to the closest measure of malaria exposure than previous approaches [20,22,68], however a few limitations accompanied the analysis. First, spatial effects were determined at the village level rather than location based. This may result in poor capturing of individuals’ spatial variability and cause uncertainty in estimation of model parameters and of significance levels. Secondly, due to lack of cause-specific mortality data, the effect of transmission on direct malaria mortality could not be estimated. Verbal autopsies are used to ascertain causes of death in the DSS [69,70]. The method had been criticized as it has poor sensitivity and specificity in distinguishing fevers that are caused by malaria and those that are not, especially in regions where transmission intensity has been reduced [69,71,72]. Estimation of malaria-attributable mortality from exposure-response relationships provides an alternative that, in principle, provides a better estimate of how much mortality can be reduced by malaria control.

## Conclusion

The analysis presented in this study used the most comprehensive entomological database that has been linked with vital events to assess the site-specific relationship between malaria transmission and child mortality. The relationship depends on levels of endemicity which vary considerably from site to site. It is debatable to generalize the conclusion drawn from these results to other settings as they might not be valid for areas with comparable levels of transmission, similar coverage of intervention and control programmes. However, using the INDEPTH-MTIMBA project database from several DSS in SSA [73], future works should involve conducting multisite comparison of the transmission-mortality relationship using mortality data from all sites. Assessment using other measures of transmission than EIR and evaluation of how child-specific cumulative exposure to malaria since birth, which differentiates the degree of protection against malaria among children [53,55,74-79], modifies the relationship, is necessary.

## Competing interests

The authors declare that they have no competing interests.

## Authors’ contributions

PV and TAS conceived the idea of the work, design the basis of the data analysis techniques, identified data sources, supervised the work, provided critical intellectual contents and approved the final version. SFR carried out the analysis, interpreted the results, drafted the initial manuscript and coordinated the writing. SA and HM participated in the design of the study, supervised data acquisition process, and managed and provided intellectual input for the manuscript. All authors read and approved the final manuscript.

## Supplementary Material

Additional file 1 Geostatistical model specification. Click here for file
