# Supplementary material for: Relationship between child survival and malaria transmission: an analysis of the malaria transmission intensity and mortality burden across Africa (MTIMBA) project data in Rufiji demographic surveillance system, Tanzania
Source: Malar J. 2014 Mar 28;13:124. doi: 10.1186/1475-2875-13-124 (PMC4021084; doi:10.1186/1475-2875-13-124)
Supplement: Additional file 1 — Geostatistical model specification. [file 1475-2875-13-124-S1.docx]

**Additional file 1 Geostatistical model specification**

Let $Y_{ijt}$ be the death status of a child $i$ $,i=1,2,3...n$ from a village$j$ $,j=1,2,3...v$at a calendar month $t =1,...,12$. $Y_{ijt}$is assumed to follow a Bernoulli distribution with a probability $p_{ij}$, i.e. $Y_{ijt}\sim Be(p_{ij})$ where $p_{ij}$is the probability of death. Let $\mathbf{X}_{ijt}=(X_{_{ijt}}^{(1)},X_{_{ijt}}^{(2)},X_{_{ijt}}^{(3)})$be a vector of covariates associated with $Y_{ijt}$ which are age, ITN, and EIR, respectively. We modeled the relationship between $p_{ij}$ and the covariates $\mathbf{X}_{ijt}$, the village-specific spatial $U_{j}$ and non-spatial $\varphi_{j}$, and month-specific temporal $\varepsilon_{t}=(e_{1},e_{2},...,e_{t})$random effects using a logit link via the equation $\text{logit}(p_{ij})=\mathbf{X}^{T}\mathbf{\beta}+U_{j}+e_{t}+\varphi_{j}$, where $\mathbf{\beta}$ is the vector of regression coefficients. Taking into account the measurement error of the EIR during estimation of the coefficient, the EIR (on logarithmic scale) was sampled from a normal distribution i.e. $X_{_{ijt}}^{(3)}\sim\text{N}\left(\mu_{x},\sigma_{x}^{2}\right)$where $\mu_{x}$and $\sigma_{x}^{2}$ are the mean and standard deviation of posterior predictive distribution of EIR at location $i$, $i=1,2,3...n$(a child was followed depending on the location), respectively.

The Bayesian model formulation requires specification of prior distributions for all unknown parameters. For the regression coefficients, $\mathbf{\beta}$a non-informative normal prior is adopted, i.e. $\mathbf{\beta}\sim\text{N}\left(0,10^{3}\right)$. The $U_{j}'s$, i.e. $U=\left(U(v_{1}),...U(v_{v})\right)^{T}$are assumed to derive from a multivariate normal distribution with a covariance matrix $\Sigma_{vxv}$, i.e. $\mathbf{U}\sim\text{MVN}(0,\Sigma_{vxv})$ which defines the Gaussian spatial process. The $\Sigma$ is a matrix with elements $\Sigma_{ij}$and quantify the covariance $Cov(U_{i},U_{j})$ between pair of villages $v_{i}$ and $v_{j}$ respectively. We assumed an isotropic spatial process where the spatial correlation is taken to be only a function of distance between the villages. An exponential correlation structure for the covariance matrix is adopted, that is $\Sigma_{ij}=\sigma_{sp}^{2}\exp(-d_{ij}\rho)$where $\sigma_{sp}^{2}$is the spatial variance, $d_{ij}$is the Euclidean distance between villages $v_{i}$ and $v_{j}$ and $\rho$measuring the correlation decay and also known as the effective range ($3/\rho$) and estimates the distance where the spatial correlation is less than 5%. The decay parameter $\rho$assumed to follows a gamma distribution. We modeled the $\varepsilon_{t}$ via a stationary autoregressive process of order one, i.e. $e_{1}\sim Normal(0,\sigma_{T}^{2}/(1-\gamma^{2}))$ and $e_{t}|e_{1,...t-1}\sim Normal(\gamma e_{t-1},\sigma_{T}^{2}),t\geq2$ where $\gamma$is an autocorrelation parameter $|\gamma|<1$ which adopts a bounded uniform distribution, $\gamma\sim\text{Unif}\left[-1, 1\right]$ and $\sigma_{T}^{2}$ is the temporal error [78]. The $\varphi_{j}$’s are assumed to follow a normal distribution with mean zero and a homoscedastic variance $\sigma_{e}^{2}$. Inverse gamma priors are adopted for the variance parameters $\sigma_{sp}^{2}$, $\sigma_{T}^{2}$and $\sigma_{e}^{2}$. The geostatistical models were implemented in OpenBUGS and parameters were estimated using the Gibbs sampler Markov Chain Monte Carlo (MCMC) algorithm [79]. Two parallel chains were run with a burn-in of 10000 initial samples, and the models were run till convergence before summarizing the results for statistical inferences.
